# Cardiac Rehabilitation and Endothelial Function

**DOI:** 10.3390/jcm9082487

**Published:** 2020-08-03

**Authors:** Gaetano Antonio Lanza, Michele Golino, Angelo Villano, Oreste Lanza, Priscilla Lamendola, Augusto Fusco, Massimo Leggio

**Affiliations:** 1 Fondazione Policlinico Universitario A. Gemelli IRCCS, 00168 Rome, Italy; angelo.villano.md@gmail.com (A.V.); priscilla.lamendola@policlinicogemelli.it (P.L.); 2 Department of Cardiovascular Medicine, Università Cattolica del S. Cuore, 00168 Rome, Italy; 3 Department of Heart and Vessels, Ospedale di Circolo, University of Insubria, 21100 Varese, Italy; micheleg1390@gmail.com; 4 Department of Clinical and Molecular Medicine and Psychology, Università La Sapienza, 00189 Roma, Italy; orelanza@gmail.com; 5 Fondazione Don Carlo Gnocchi IRCCS, 20147 Milan, Italy; afusco@dongnocchi.it; 6 Cardiology Operative Unit, San Filippo Neri Hospital, 00135 Rome, Italy; mleggio@libero.it

**Keywords:** cardiac rehabilitation, endothelial function, clinical outcome

## Abstract

Endothelial dysfunction is an early abnormality in the process of atherosclerosis and cardiovascular disease and has been associated with worse clinical outcome. Cardiac rehabilitation (CR) has been reported to be helpful to reduce cardiovascular events in various types of cardiac disease, but the mechanisms of its beneficial effects remain only partially known. In this article, we review the studies that assessed the effect of CR on endothelial function in patients with various cardiac conditions. Available data show that CR significantly improves impaired endothelial function in these patients, which may contribute to the beneficial effects of CR on clinical outcome.

## 1. Introduction

Cardiac rehabilitation (CR) is a program of physical and respiratory exercises, handled by an interdisciplinary team, aimed to improve cardiovascular function and physical capacity mainly after an acute cardiac event or intervention. CR programs have been reported to decrease morbidity and improve quality of life in these patients; furthermore, they have also been suggested to improve survival. These beneficial effects are likely mainly achieved through a tighter control of cardiovascular risk factors (CVRFs) as well as adherence to optimal medical therapy, which prevent the progression of atherosclerosis and reduce complications of atherosclerotic lesions [1,2,3,4,5]. CR programs, indeed, mainly focus on exercise training, but appropriate treatment of CVRFs, optimization of medical therapy, psychosocial interventions and patients’ education also constitute crucial integrative measures [5,6,7,8,9].

Based on the evidence of their beneficial cardiovascular effects, CR has a Class I indication in several American and European guidelines for patients with various heart conditions, including heart surgery, acute myocardial infarction, stable angina, heart failure (HF) with reduced left ventricular function, as well as peripheral artery disease [10,11,12,13,14]. Yet, despite the beneficial properties of CR have widely been documented and are widely accepted, the mechanisms through which it achieves its clinical benefits are still incompletely known.

This article is mainly focused on briefly reviewing the evidence of a favorable effect of CR programs on endothelial function in cardiac patients. An impairment of endothelial function is indeed considered a major cause of susceptibility to vascular complications and an improvement in endothelial function may therefore constitute a major mechanism through which CR may improve vascular function and, consequently, clinical outcome. We will precede the review of the available data on the effects of CR on endothelial function by a comment on the function of the normal endothelium and the implications of its dysfunction and by a short review of the main methods applied to assess endothelial function in clinical studies.

## 2. Endothelial Function and Dysfunction

The vascular endothelium has an outstanding role in maintaining vascular homeostasis and regulating blood vessel function. Thus, in normal people, the endothelium is a major regulator of blood flow through its vasodilator properties. These are mainly exerted by the release of nitric oxide (NO), but also of prostacyclin (PGI_2_) and endothelium-derived hyperpolarizing factor (EDHF), in response to various chemical substances (e.g., bradykinin, acetylcholine, serotonin), but also physical stimuli (e.g., shear stress). Furthermore, the normal endothelium also exerts anti-platelet, anti-coagulant, fibrinolytic, anti-inflammatory and antiproliferative actions [15]. Again, endothelial NO production and release play a major role in these beneficial effects, in particular in preserving the anti-thrombogenic property of the endothelial lining [15].

Accordingly, a dysfunctional endothelium is associated with a reduction of these beneficial effects. An impairment of NO production is an early consequence of endothelial cell dysfunction. The reduced NO release by endothelial cells results in an increase of basal vascular tone and in a reduced dilator response to stimuli that exert their vascular dilator effect by inducing NO release from endothelial cells. Of note, a dysfunctional endothelium may also display prevalent vasoconstrictor effects, due to the increased release of vasoconstrictor agents, such as endothelin-1. Furthermore, the impaired NO release also results in a reduction of the protective antithrombotic and anti-inflammatory effects of the endothelium. Moreover, the dysfunctional endothelium may also assume pro-aggregate and proinflammatory properties [16].

These changes in endothelial cell function are now considered the first step through which CVRFs lead to the formation of atherosclerotic plaques [17,18,19,20]. All CVRFs, indeed, have been shown to cause endothelial cell dysfunction, mainly through noxious chemical stimuli, including modified lipoproteins, constituents of cigarette smoke, high glucose levels and inflammatory cytokines. These factors induce the synthesis and expression on endothelial cell surface of adhesion molecules (e.g., E-selectin, vascular cell adhesion molecule [VCAM-1]), procoagulant substances (e.g., tissue factor) and proinflammatory chemokines (e.g., interleukin-8), and eventually cause endothelial cell injury that results in focal endothelial cell desquamation. This triggers platelet adhesion and aggregation with local release of growth factors, which induce migration, proliferation and changes of smooth muscle cells that eventually result in the formation of fibromuscular atherosclerotic plaques [21]. Importantly, both functional and structural changes similar to those described for the abnormal chemical stimuli can be also caused by exposure to disturbed blood flow, as it may occur in hypertension. Turbulent flow, indeed, seems the be able to activate important pathophysiological endothelial genes (such as those for platelet derived growth factor and VCAM-1).

In agreement with its deleterious effects, endothelial dysfunction has been shown to predict cardiovascular events in various clinical settings, including patients with CVRFs, those with acute or chronic coronary artery disease (CAD) and HF. Of note, the prognostic implications of endothelial dysfunction have been reported for both coronary and peripheral, as well as macrovascular and microvascular, circulation [22,23,24,25,26,27,28,29].

Importantly, the control of cardiovascular risk factors with some drugs, such as statins and antihypertensive drugs, improves endothelial function [30,31], which likely contributes to their beneficial clinical effects. Among non-pharmacological therapy, aerobic exercise training has been shown to improve endothelial function in patients with CVRFs, including hypertension and diabetes, both in the large arteries and microcirculation [32,33]. Aerobic exercise may exert its beneficial effects by modifying the pattern of flow at the level of arterial branch points, leading to less turbulent flow, which would favor the expression of atheroprotective genes, such as NO synthase (eNOS), with the restoration of a vasodilator and vasoprotective phenotype of the endothelium [33]. Of note, also an increase of other protective factors, such as PGI_2_ and EDHF may contribute to the improved physiologic effects of the endothelium by exercise [34,35]. The improvement of autonomic tone, a reduction of inflammation and oxidative stress all may contribute to the positive effects of exercise on endothelial cell function [33,34,35].

## 3. Assessment of Endothelial Function

Several methods have been described and are used to assess the functional state of the endothelium. Most of these methods are devoted at exploring systemic endothelial function through the assessment of endothelium-mediated dilator function both in large peripheral arteries and microcirculation and include flow-mediated dilatation (FMD), venous occlusion plethysmography, peripheral arterial tonometry (PAT) and laser Doppler flowmetry. Furthermore, indications on the functional state of the vascular endothelium can be obtained from in vivo or in vitro measurements of markers of endothelial activity.

Importantly, methods have also been developed to specifically assess endothelial function in the coronary circulation, although their application in clinical practice is challenged by the need of invasive investigation. Yet, in cardiac diseases it would be important to specifically assess coronary, rather peripheral, endothelial function, as the relation between the presence and severity of endothelial dysfunction in peripheral and coronary circulation has been shown to be less than optimal [36,37].

A detailed review of the methods applied to assess endothelial function can be found elsewhere [38,39,40]. In the next paragraphs, we will only briefly review the methods that have most often been used in clinical studies on the effects of CR programs on endothelial function.

### 3.1. Peripheral Circulation

#### 3.1.1. Flow-Mediated Dilatation

FMD has become one of the most used techniques to assess systemic endothelial function non-invasively. It consists in measuring by ultrasound the diameter change of a peripheral artery in response to a maximal increase of blood flow consequent to reactive hyperemia. The increase in blood flow velocity, indeed, leads to stimulation of endothelial cells to release NO by the increased shear stress, which results in the dilatation of the vessel.

**Figure 1:**
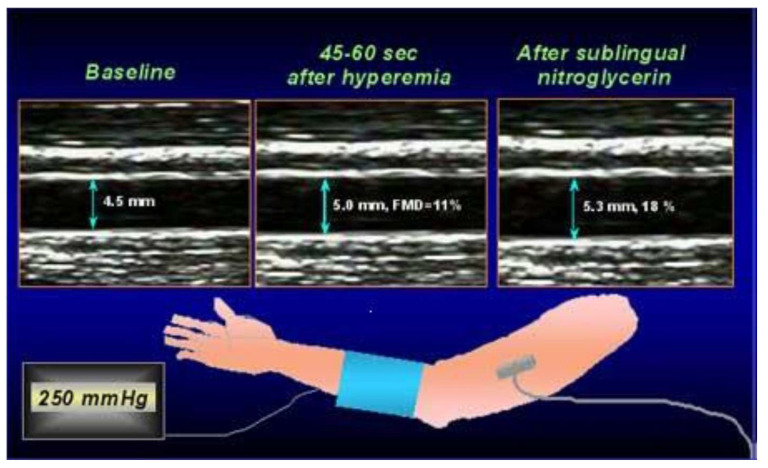

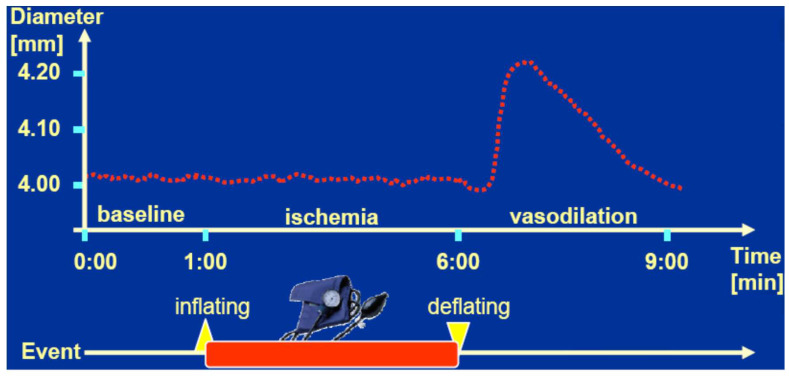
Illustration of the method to assess flow-mediated dilation (FMD). Brachial artery diameter is measured at baseline and during hyperemia consequent to 5-min forearm ischemia; FMD is calculated as the percent increase of the artery diameter during hyperemia compared to baseline.

In clinical practice, FMD is usually assessed in a brachial artery (Figure 1). To this scope, images of the artery are first obtained at rest by a high frequency (10 MHz) probe attached to a high-resolution ultrasound system machine and basal diameter of the vessel is measured. Then, a forearm cuff, positioned one centimeter under the antecubital fossa, is inflated at 50 mmHg above the systolic blood pressure, thus inducing forearm ischemia. The cuff is quickly released after five minutes of ischemia to elicit forearm reactive hyperemia. FMD is then calculated as the maximum percent change of the brachial artery diameter during hyperemia compared with the basal diameter.

FMD values obtained manually by operators are subject to some variability of the measurements, which affects the comparability of the data and has until now limited a wide applicability in the clinical setting and precluded a recommendation of its use in clinical guidelines [41]. The use of a mechanical support to maintain the probe in a fixed position and the use of software that automatically detect the vessel edges and allow continuous measurements of the vessel diameter throughout the entire examination improve the reproducibility of the data and may allow appropriate standardization of the method and wider applicability both in the research and clinical field [42,43].

FMD has been reported to be impaired in several clinical conditions, including subjects with various CVRFs, patients with stable CAD or HF, as well as in acute settings of CAD [38,44]. Furthermore, FMD has been shown to predict cardiovascular events, particularly in patients with established cardiovascular diseases or at high cardiovascular risk [45,46,47].

Measurement of forearm blood flow velocity at baseline and during reactive hyperemia may also give some insight in the status of peripheral microcirculation. A limited decrease of peripheral resistances during hyperemia, indeed, may indicate an impairment of the microvascular dilatation function, which may also involve the endothelium. Accordingly, an impairment of FMD may, in fact, depend on a reduced microvascular dilatation, which limits forearm blood flow increase, rather than an impaired NO-mediated dilatation of the brachial artery.

#### 3.1.2. Peripheral Arterial Tonometry

**Figure 2:**
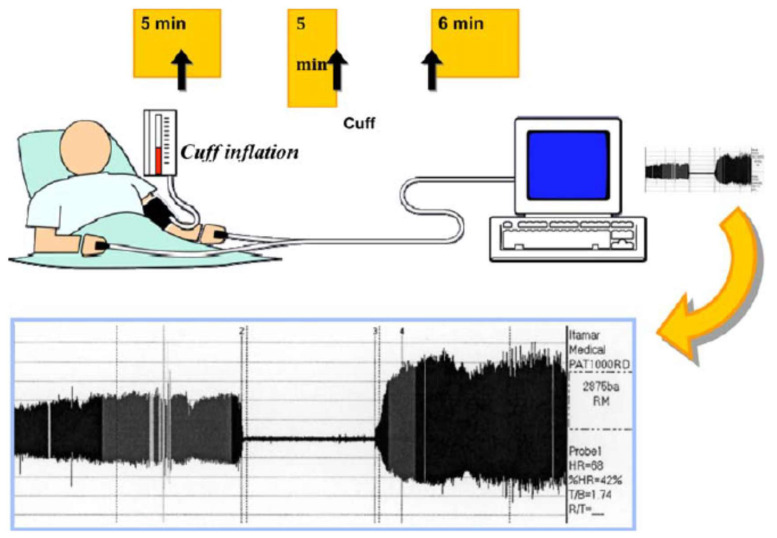
Illustration of the method of peripheral arterial tonometry. Modified from [40].

This technique investigates peripheral microcirculatory dilatation by measuring pulsatile arterial volume changes by finger plethysmography (EndoPAT) (Figure 2). Practically, a pressure cuff is placed on a forearm and inflated to suprasystolic pressure levels to produce five minutes of ischemia, followed by reactive hyperemia after cuff deflation. A pneumatic probe applied to the fingertip of the same arm, by recording the digital pulse wave amplitude (PWA), allows measuring the changes in arterial blood volume at baseline and during hyperemia. The increase in blood flow during hyperemia is expressed by the reactive hyperemia index (RHI), i.e., the ratio between the post- and preocclusion PWA values. The RHI is normalized by the measurement of the opposite arm, which serves as control of the effect of hyperemia.

RHI is considered to reflect microvascular endothelial function [48] However, digital microvessel dilatation during hyperemia is only in part dependent on NO availability [49], whereas it is rather a marker of comprehensive peripheral microvascular capacity. RHI has been found decreased in patients with CVRFs [50,51] and may also predict cardiovascular events, similarly to FMD [52]. However, a poor correlation between RHI and FMD has been found in various settings, suggesting that the two methods explore different, although complementary, aspects of the vascular function [53,54]. The reproducibility of PAT has been reported to be rather low, which makes difficult to standardize its application in clinical practice [55,56], and has until now also precluded its inclusion in clinical guidelines [41].

#### 3.1.3. Laboratory Markers

The functional state of the endothelium can also be derived by measuring blood concentrations of substances that are typically or mainly, released by endothelial cells following endothelial activation and dysfunction, as previously described. An increase in the blood of a large number of biomarkers reflecting endothelial activation, in particular in CAD populations, has been described [39], but those most frequently used in clinical studies include some soluble cell adhesion molecules, as intercellular adhesion molecule-1, VCAM-1, E-selectin and the von Willebrand factor. Serum biomarkers are rather easy to be obtained, but a large variability and the multiple factors that influence their concentrations may limit their clinical applicability.

More sophisticated laboratory methods to assess functional endothelial state include the measurement of endothelial progenitor cells (EPCs) concentrations, which are crucial for repairing endothelial lesions [57], and the development in vitro of cultures of endothelial progenitor cells [58]. These methods, however, are poorly available and may present various technical issues.

### 3.2. Coronary Circulation

In coronary circulation, endothelial function can adequately be assessed only by invasive methods. Although other stimuli may be used (e.g., atrial pacing, serotonin, substance P), at present it is usually assessed by intracoronary administration of low to medium doses of acetylcholine, which in normal subjects dilate coronary vessels by inducing NO release from endothelial cells [59]. The endothelium-mediated dilator effect of Ach infusion on epicardial vessels is obtained by measuring the changes in vessel diameter by quantitative coronary angiography or intravascular ultrasound. The effect on coronary microcirculation is obtained by measuring coronary blood flow (CBF) by either intracoronary Doppler recording of blood flow velocity or the thermodilution method [60,61]. An increase of 50% or more of CBF is considered as normal.

An alternative stimulus to assess endothelial function in coronary circulation is cold pressor test (CPT), which is performed by putting a hand in ice for 90–120 s. In normal subjects, CPT induces a mild adrenergic activation which results in NO release through alpha–receptor stimulation on endothelial cells [62]. In dysfunctional vessels, however, CPT may trigger sympathetic-mediated vascular constriction [63,64]. Several studies have assessed coronary microvascular endothelial function by assessing the CBF increase in response to CPT, also using non-invasive methods (e.g., positron emission tomography, cardiac magnetic resonance and transthoracic Doppler recording) [55]. The reliability of this method, however, is limited by the impossibility to exclude some direct coronary constriction in the limitation of CBF increase.

## 4. Cardiac Rehabilitation and Endothelial Function

CR programs mainly focus on exercise training, but they involve a careful implementation of integrative measures, including appropriate treatment of CVRFs, optimization of medical therapy, psychosocial interventions and patients’ education [1,2,3,4,5,6,7,8,9]. CR programs, indeed, involve three phases: (1) an in-hospital phase, in which patient’s health situation and goals, education and cardiovascular risk factors are discussed by a multidisciplinary medical team; (2) a second phase, which can be performed in a residential (inpatient) setting or an ambulatory (outpatient) setting, in which the patient undergo a program of exercises, aggressive risk-factor modifications and education classes; (3) a long-term maintenance phase, in which the patient is encouraged to continue to maintain optimal control of cardiovascular risk factors and to practice an individualized, but regular physical activity [65]. CR rehabilitation programs are therefore expected to improve endothelial function through multiple mechanisms. The emphasis and surveillance on the tight control of CVRFs, diet and adherence to medical therapy certainly play a relevant role to this scope and in the beneficial effects on clinical outcome of CR programs, in particular in CAD patients, even when treated with contemporary evidence-based forms of therapy [66,67]. However, as discussed in the paragraph on endothelial function, aerobic exercise programs may add further favorable effects in cardiac patients [1,2,3].

Several studies have investigated the effects of CR programs on endothelial function, mainly in those recovering from an acute myocardial infarction (AMI), but also in those with stable CAD or HF. A summary of the main characteristics and results of these studies is shown in Table 1. 

## 5. Acute Myocardial Infarction

Several studies investigated the effects of CR programs on endothelial function in patients recovering from AMI. Vona et al. [68], randomized 52 patients with a recent uncomplicated AMI to CR, consisting of moderate aerobic training on a cycle ergometer three times per week or usual care. FMD, CPT and nitrate-mediated dilation (NMD) were assessed at baseline and after 3 months of treatment. While no changes in NMD were observed in both groups, a significant greater improvement of FMD was found in the CR group (from 1.66% ± 4.11% to 9.39% + 4.87%) compared to controls (from 2.04% + 3.4% to 4.4% + 3.9%; *p* < 0.01). Of note, CPT caused a constriction of the brachial artery at baseline in both groups; at follow-up, however, no changes in brachial artery diameter was observed in the CR group, whereas vasoconstriction still occurred in controls. The improvement of endothelial function was associated with a significant increase in exercise tolerance. Importantly, these benefits of CR disappeared in the treated group after detraining, suggesting that continuous exercise training may be necessary to maintain the improvement of endothelial function.

Lee et al. [69] randomized 81 patients with AMI or coronary revascularization to comprehensive hospital-based or home-based cardiac rehabilitation. FMD was assessed at baseline and after three months of a CR program, together with 24-h ambulatory blood pressure monitoring and measurement of plasma levels of von Willebrand factor, D-dimer, fibrinogen, soluble P-selectin and plasma viscosity. A significant increase in FMD was observed in the whole group (from 4.16% [1.46–7.42%] to 6.9% [2.34–11.5%]; *p* < 0.001), together with a significant reduction of plasma levels of vWF. No changes in FMD and plasma markers of endothelial function were observed in 20 matched control patients who did not undergo CR. No significant differences in endothelial function improvement were found between home-based and hospital-based cardiac rehabilitation programs.

Peller et al. [70] assessed reactive hyperemia by PAT in 29 patients with AMI before and after CR, started at least four weeks after AMI and consisting of 12 or 24 rehabilitation sessions (3–4 per week) according to patient’s preference. At the end of CR, the lnRHI increased from 0.49 (0.36–0.67) to 0.58 (0.43–0.74), but the difference did not achieve statistical significance (*p* = 0.14). However, in the subgroup of patients with endothelial dysfunction at baseline (*n* = 16 or 55.2%), as diagnosed by an lnRHI lower than 0.51, lnRHI improved significantly at follow-up from 0.37 (0.27–0.42) to 0.53 (0.43–0.66) (*p* = 0.002), thus suggesting that CR may have beneficial effects on endothelial function only when it is significantly depressed at baseline. However, patients training for 24 sessions (*n* = 16) had similar lnRHI changes to those of patients training for 12 sessions (*n* = 9; *p* = 0.44).

Finally, Oliveira et al. [71] randomized 96 AMI patients to an 8-week exercise-based CR program, consisting of aerobic exercise at 70–85% of maximal heart rate during three weekly sessions or controls. Markers of endothelial dysfunction, arterial stiffness, investigated through carotid-femoral pulse wave velocity (cf-PWV), and markers of inflammation were assessed at baseline and at the end of treatment. No significant changes were found between groups in endothelial dysfunction biomarkers, as well as cf-PWV and inflammatory biomarkers, although a decrease of cf-PWV was noticed in patients who attended at least 80% of exercise sessions. In this study, however, the exercise program was relatively short and only adhesion molecules VCAM-1 and ICAM-1 were measured as markers of endothelial function.

## 6. Stable Coronary Artery Disease

In patients with clinically stable CAD, the effects of CR programs on endothelial function were investigated by a few studies. Cornelissen et al. [72] assessed FMD and RHI by PAT at baseline and after a 12-week cardiac rehabilitation program in 146 patients with stable CAD. FMD showed a significant improvement (average, from 10.0% to 13.0%; *p* < 0.001), whereas no significant change was observed in RHI (average, from 1.92 to 1.96; *p* = 0.47).

Edwards et al. [73] assigned 18 stable CAD patients to a 12-week standard CR program or control. Endothelial function was assessed by FMD at baseline and at the end of CR. FMD increased from 7.9% ± 2.2% to 11.1% ± 3.1% (*p* < 0.05) in the CR group but showed no change in the control group (8.5% ± 2.3% at baseline and 8.2% + 2.3% at follow-up). In addition, an increase in plasma nitrite and nitrate levels was observed in the CR group, confirming the improvement of endothelial function. Moreover, an increase of superoxide dismutase levels, an antioxidant enzyme, was found in the CR group, but not in controls, suggesting that the favorable effects of endurance exercise training as part of a CR program on endothelial function may be related to decreased oxidative stress.

Gokce et al. [74] assessed the effects of a 10-week supervised CR exercise program with moderate leg intensity exercise of FMD on both brachial artery and posterior tibial artery of 40 stable CAD patients. Exercise training was conducted at an intensity of 45% to 85% of heart rate reserve, derived from an initial exercise test and performed 30–40 min three times/week. Data were compared with those of a control group of 18 matched CAD patients with a sedentary lifestyle. Exercise was associated with an increase in functional capacity and significant improvement in FMD in the posterior tibial artery (from 9.7% ± 2.1% to 11.7% ± 2.1%) compared to controls (from 9.9% ± 2.1% to 9.1% ± 2.1%; *p* < 0.05). No significant difference in the changes of FMD was observed between the two groups in the brachial artery (*p* = 0.23), thus suggesting that exercise may improve endothelial function only in peripheral arteries of the exercised limbs. However, FMD in the brachial artery improved by 1.9 points in CR patients (6.4% ± 3.4% vs. 8.3% ± 3.4%), but only by 0.3 points in controls (7% ± 3.5% vs. 7.3% ± 3.5%), suggesting that the lack of statistical difference in brachial FMD between the two groups could be related to a limited statistical power of the study.

Gagliardi et al. [75] randomized 21 patients with chronic stable CAD to a rehabilitation program with three exercise bouts weekly over 12 weeks (11 patients) or no rehabilitation program (10 patients). They measured EPCs and vascular endothelial growth factor (VEGF) at baseline. A significant reduction of EPCs and an increase in VEGF at one and three months of follow-up was found, suggesting improvement of endothelial function, but no differences were observed between the two groups.

The multicenter SAINTEX study compared the effects of aerobic interval training (AIT) and aerobic continuous training (ACT) on peak VO_2_, FMD, CVRFs and quality of life in patients with stable CAD [76]. In this trial, 200 patients were randomized to a supervised 12-week CR program of three weekly sessions of either AIT (90–95% of peak HR) or ACT (70–75% of peak HR) on a bicycle. A similar improvement of FMD was observed in the two groups, together with a similar improvement of peak VO_2_, CVRFs and quality of life. The improvement of FMD showed a significant correlation with the peak VO_2_ (*r* = 0.17; *p* = 0.035). No effects of both types of ET programs were observed on the blood levels of EPCs, angiogenic T cells and endothelial microparticles [77].

While the previous studies investigated peripheral endothelial function, in a study Hambrecht et al. assessed coronary endothelial function [78]. They randomized 19 patients with documented obstructive CAD and coronary endothelial dysfunction, defined as either epicardial vasoconstriction or diameter dilation < 5% in response to Ach, to an exercise-training group (10 patients) or a control group (9 patients). The coronary response to increasing doses of intracoronary acetylcholine (0.072, 0.72 and 7.2 μg per minute) was assessed at baseline and after four weeks of treatment. The diameter of epicardial coronary vessels and mean peak flow velocity were measured by quantitative coronary angiography and Doppler velocimetry, respectively. At baseline, the two groups showed similar responses to acetylcholine. At the follow-up study the responses to Ach remained unchanged in the control group. In the exercise training group, however, a significant reduction of the vasoconstrictor effect of Ach on epicardial vessels was observed (diameter reduction of 15.2% ± 2.2% vs. 6.8% ± 2.5% at the maximal Ach dose; *p* for change vs controls < 0.05). Mean peak flow velocity in response to Ach in the exercise group showed a significant improvement (from 78.1 ± 15.5 vs. 141.6 ± 27.7 cm/s at the highest dose; *p* for change < 0.05). Thus, this study showed that exercise training improves endothelial function both in epicardial and resistance coronary vessels in patients with coronary artery disease.

## 7. Heart Failure

Several studies have shown that an abnormal endothelial function is present in patients with HF [81,82], likely as a result of the activation of the adreno–medullary neuro–humoral axis. Endothelial dysfunction in HF patients may cause increased vascular resistances and decreased organ perfusion, leading to exercise intolerance and contributing to HF progression. Some studies, indeed, have reported that endothelial dysfunction in HF patients is associated with a worse clinical outcome [83,84]. Accordingly, an improvement of endothelial function may favor the regulation of organ blood flow, improve physical performance and delay the progression of the disease. Exercise-based CR programs have been shown to improve exercise capacity and quality of life in HF patients [85] and an improvement of endothelial function might contribute to these favorable effects.

Two studies investigated the effects of CR on endothelial function of patients with HF. Tanaka et al. [79] retrospectively reviewed the effect of 5-month CR on endothelial function, as assessed by brachial FMD, in 30 patients with HF with reduced left ventricle ejection fraction and 30 patients with HF with preserved ejection fraction. FMD did not improve significantly at follow-up in both groups. CR improved exercise capacity, however, only in patients who, at baseline, showed endothelial dysfunction (defined as FMD ≤ 5%), but this seemed independent of significant changes in FMD values.

Legallois et al. [80] assessed the effect of CR on coronary endothelial function in 29 clinically stable patients with HF related to non-ischemic dilated cardiomyopathy. They measured myocardial blood flow (MBF) by positron emission tomography at rest and during CPT at baseline and after 12 weeks of a CR program, including 36 supervised exercise sessions, diet therapy, medical advice, patient education and medical therapy optimization. At baseline, MBF showed no changes during CPT (1.16 ± 0.41 vs. 1.14 ± 0.31 mL/min/g, respectively). After the completion of the CR program, MBF increased significantly at rest (1.31 ± 0.38 mL/min/g; *p* = 0.04) and after CPT (1.51 ± 0.38 mL/min/g; *p* < 0.001) compared to basal values. Furthermore, after CR, MBF increased significantly in response to CPT, whereas no significant change had been observed at the baseline test (0.19 ± 0.22 vs. −0.03 ± 0.22 mL/min/g, respectively; *p* < 0.001).

## 8. Conclusions

In conclusion, although with some variable results, the complex of available data supports the notion that, in cardiac patients, CR programs improve endothelial function, particularly when it is significantly impaired. This beneficial effect is likely mediated by a combination of factors, including exercise training programs, lifestyle changes and a tighter control of CVRFs through a better adherence to medical therapy.

The improvement of endothelial function found in the published studies seems largely independent of the type of aerobic exercise training programs, including walking on treadmill vs. bicycle, as well as continuous vs. interval training. Thus, rather than recommending for specific exercise programs, what seems important to achieve beneficial effects on the cardiovascular system and endothelial function, is that the supervised exercise training allows to reach an adequate exercise intensity (e.g., 70–80% of maximal HR for 30 min) and that sessions are performed on a regular basis (e.g., three times per week) and for an adequate period (e.g., 12 weeks). The beneficial effects of CR in cardiovascular patients, indeed, strongly depend on the volume and intensity of exercise program delivery [86]. Importantly, the maintenance of an adequate physical activity after the end of the supervised ET in CR programs is crucial to also maintain the improvement of endothelial function, although the optimal frequency, type and intensity of long-term physical activity remains to be established.

According to available data, the assessment of endothelial function could, therefore, be useful to assess whether CR programs result in benefits on vascular function in individual patients. To this scope, FMD (with forearm blood flow assessment) seems the most simple and comprehensive method, but it still suffers from center and operator variability; efforts should be done, therefore, to achieve a standardization of this (as well as other) method for endothelial function assessment that may allow a large application in clinical practice.

Importantly, while an improvement of endothelial function by CR programs in cardiac patients has been suggested by several studies and is expected to portend beneficial effects on cardiovascular complications, whether it actually translates into improved clinical outcome remains to be demonstrated. To this scope, adequately designed and sized randomized multicenter clinical trials should be planned, using both standardized CR exercise training programs and methods for the assessment of endothelial function.

## Figures and Tables

**Table 1:** Summary of the main studies that assessed the effects of cardiac rehabilitation (CR) on endothelial function.

Study	Population	No. Patients	Study Design	Assessment of ED	Exercise Program	Results
Vona [68]	Recent AMI	52	RCT	FMD, CPT	3 months of moderate aerobic ET	CR significantly improved ED vasodilation (*p* < 0.01)
Lee [69]	Previous AMI or coronary revascularization	81	RCT	FMD, vWF	3 months of home/hospital-based CR program	CR improved FMD and reduced vWF (all *p* ≤ 0.001)
Peller [70]	Recent AMI	25	Prospective uncontrolled	RHI-PAT	4 weeks (12–24 ET session)	CR did not significantly improve endothelial function (*p* = 0.14)
Oliveira [71]	Recent AMI	96	RCT	Endothelial biomarkers	8 weeks of aerobic ET at 70–85% of maximal HR during 3 weekly sessions	CR did not reduce the markers of ED
Cornelissen [72]	Stable CAD	146	Prospective uncontrolled	FMD, RHI-PAT	12 weeks (3 sessions per week at an intensity of 80% of HR reserve	CR improved FMD (*p* < 0.0001), but not RHI (*p* = 0.47)
Edwards [73]	Stable CAD	18	Prospective controlled	FMD, nitrites, nitrates	12 weeks (3 times per week of treadmill walking and stationary cycling at an intensity of 40–50%, to a maximum 70–85% of HR reserve.	CR improved FMD and increase nitrate and nitrite levels
Gocke [74]	Stable CAD	58	Prospective controlled	FMD	10 weeks of leg exercise of moderate intensity (30 min 3 times per week)	CR improved FMD (*p* = 0.02), in particular in the exercised limbs
Gagliardi [75]	Stable CAD	21	RCT	VEGF, EPCs	12 weeks (three weekly exercise bout)	CR determined a reduction of EPC and an increase in VEGF
SAINTEX-CAD study [76,77]	Stable CAD	200	RCT	FMD, EPCs	12 weeks of aerobic interval vs. continuous ET on a bicycle	Both ET programs improved FMD, but had no effect on EPCs
Hambrect [78]	Stable CAD	19	RCT	Coronary epicardial and MV response to Ach	4 weeks (10 min 6 time a day on a bicycle ergometer to the 80% of HR)	CR improved epicardial and MV endothelial response to Ach
Tanaka [79]	HFrEF/HFpEF	78	Retrospective study	FMD	5 months (2–3 times per week of training on a cycle ergometer for 20 min at anaerobic threshold level)	CR did not improve FMD, but improved exercise capacity in patients with ED at baseline
Legallois [80]	HF in DCM	29	Prospective study	MBF response to CPT	12 weeks (36 aerobic ET sessions on the basis of ventilatory threshold)	CR improved MBF response to CPT (*p* < 0.001)

Ach—acetylcholine; AMI—acute myocardial infarction; CAD—coronary artery disease; CPT—cold pressor test; CR—cardiac rehabilitation; DCM—dilated cardiomyopathy; ED—endothelial dysfunction; EPCs—endothelial progenitors cells; ET—exercise training; FMD—flow mediated dilation; HF—heart failure; HFpEF—HF with preserved ejection fraction; HFrEF—HF with reduced ejection fraction; HR—heart rate; MBF—myocardial blood flow; MV—microvascular; RCT—randomized controlled trial; RHI-PAT-reactive hyperemia index on peripheral arterial tonometry; VEGF—vascular endothelial growth factor; vWF—von Willebrand factor.
